# Design of a Two-level Adaptive Multi-Agent System for Malaria Vectors driven by an ontology

**DOI:** 10.1186/1472-6947-7-19

**Published:** 2007-07-02

**Authors:** Guillaume Koum, Augustin Yekel, Bengyella Ndifon, Josiane Etang, Frédéric Simard

**Affiliations:** 1 Laboratoire de Recherche en Informatique, Multimédia et Applications, Ecole Nationale Supérieure Polytechnique. B.P. 8390 Yaounde, Cameroon, Africa; 2 Institut de Recherche pour le Développement -Unité de Recherche 016, Organisation de Coordination pour la lutte contre les grandes Endémies en Afrique Centrale. B.P 288 Yaounde, Cameroon, Africa; 3 Institut de recherches Médicales et d'études sur les Plantes Médicinales, Ministère de la Recherche Scientifique et de l'Innovation. B.P 6163 Yaounde, Cameroon, Africa

## Abstract

**Background:**

The understanding of heterogeneities in disease transmission dynamics as far as malaria vectors are concerned is a big challenge. Many studies while tackling this problem don't find exact models to explain the malaria vectors propagation.

**Methods:**

To solve the problem we define an Adaptive Multi-Agent System (AMAS) which has the property to be elastic and is a two-level system as well. This AMAS is a dynamic system where the two levels are linked by an Ontology which allows it to function as a reduced system and as an extended system. In a primary level, the AMAS comprises organization agents and in a secondary level, it is constituted of analysis agents. Its entry point, a User Interface Agent, can reproduce itself because it is given a minimum of background knowledge and it learns appropriate "behavior" from the user in the presence of ambiguous queries and from other agents of the AMAS in other situations.

**Results:**

Some of the outputs of our system present a series of tables, diagrams showing some factors like Entomological parameters of malaria transmission, Percentages of malaria transmission per malaria vectors, Entomological inoculation rate. Many others parameters can be produced by the system depending on the inputted data.

**Conclusion:**

Our approach is an intelligent one which differs from statistical approaches that are sometimes used in the field. This intelligent approach aligns itself with the distributed artificial intelligence. In terms of fight against malaria disease our system offers opportunities of reducing efforts of human resources who are not obliged to cover the entire territory while conducting surveys. Secondly the AMAS can determine the presence or the absence of malaria vectors even when specific data have not been collected in the geographical area. In the difference of a statistical technique, in our case the projection of the results in the field can sometimes appeared to be more general.

## 1-Background

Malaria affects hundred of millions of people in the world, particularly in under developed countries. Recent studies carried out show that, malaria is the leading cause of morbidity and mortality. Death rate remains particularly high among children and pregnant women, especially from poor backgrounds. In Cameroon, statistics also show that the disease has often reduced the life expectancy to 49 years, with devastating impact on the economy [1] Statistics indicate that malaria accounts for 40–50 per cent of medical consultations in hospitals nationwide. This situation is not only due to the increasing resistance of malaria parasite and its vectors, but the lack of awareness on the part of communities to fight the disease. Against these backdrops, actions must be taken because prevention is obviously better than a cure. Prevention oriented activities are efficient to curb the malaria pandemic. In this view, vector control is a cornerstone of the strategy that needs to be implemented and monitored [2]. This is one of the public health priorities.

On a computational point of view, the health infrastructure can be a widely distributed network in a country. The data is being generated at the local level, compiled and sent to national level successively through next higher administrative unit and by the time when reaching policy makers it loses its significance. Therefore, an efficient information management system is highly required for surveillance of diseases and control strategies [3]

The purpose of this article is to show how an Adaptive Multi-Agent System (AMAS) functions in the field of malaria transmission. Our case is that of heterogeneous agents which must cooperate and be integrated [4-6]. Agents integration architecture permits a heterogeneous, distributed set of agents working together to address complex problems. Unfortunately, integrating software agents and humans to perform real-world tasks in many domains remains difficult. The challenge is that we need a system working in an operational level and in a decision-making level. So agents must cooperate in each level and levels must also cooperate each other. We are then interested in the following issues: the cooperation structure, the roles and responsibilities held by components within the cooperation, the flow of information within the cooperation, the capabilities required or available within the cooperation, and the context in which the cooperation holds [7]

We can compare our system to Biomapper[8] which is a kit of GIS and statistical tools designed to build habitat suitability (HS) models and maps for any kind of animal or plant. It is centered on the Ecological Niche Factor Analysis (ENFA) that allows to compute HS models without the need of absence data. However our AMAS because of the presence of an Expert System Agent and the Ontology which link to the two part of the system is more flexible. It is oriented towards a qualitative data aspect compared to the statistical approach which is merely quantitative

The plan of the article follows. After our introduction in the first section, the AMAS is presented in the second section as well as the agents community and communication. It draws the framework of our system. The third section intends to show some results with examples related to the study. The fourth section deals with a short discussion. The fifth section concerns the conclusion.

## 2-Methods

### 2.1-Adaptive MAS (AMAS)

Our solution for an information system as far as malaria vectors control is concerned, in order to fight the illness, is a two-level Adaptive Multi-Agent System (AMAS) [9]. The low level part is constituted of organization agents. Through this part of the AMAS, it is possible to build up mechanisms of fight against malaria vectors by paying attention on natural settings of vectors to destroy them. The high level part comprises analysis agents. It offers framework to define plans which aim at preventing, rather than striving to cure malaria.

### Definitions

#### Definition 1

An adaptive MAS (AMAS) is a system [10]

1) 1- opened in a systemic sense of information transfer, to the environment submitted to fundamental trends;

2) which publishes information it receives : it has a transcription/restitution interface that permits it to communicate with the environment;

3) which apprehends its universe (environment and itself) and represents the environment states, and that of its proper organization by designing action plans adapted to its contextual situation;

4) which continuously modifies its structure while functioning;

5) which alters under the pressure of fundamental trends and which by necessity must maintain itself in an adaptativity state.

#### Definition 2 [10]

An adaptive agent is an agent of adaptive system capable to generate and interpret implicitly indications concerning fundamental trends of the system. These indications have implicit influence on its state and its structure whereas it operates as an original agent with respect to its actions, pro-actions, and communications.

### 2.2-Agents community

The technical aspects of our solution involve three organization agents at the first level: a Database Management System (DBMS) Agent, a Geographic Information System (GIS) Agent and an Expert System (ES) Agent. All those agents cooperate through another organization agent which is a User Interface (UI) Agent. At the second level are implemented the following analysis agents: a Data Mining (DM) Agent, a Decision Support System (DSS) Agent. These agents can have access directly to the UI Agent.

The DBMS Agent is an agent taking the place of a DBMS as a collection of stored operational data used by the application systems. We consider our DBMS to be a relational or an object-oriented one.

The GIS Agent represents a GIS which is an assembly designed to efficiently acquire, store, manipulate, retrieve, analyze, display and report all forms of geographically referenced information geared towards a particular set of purposes [11].

The Interface Agent which indirectly manages human-computer interfaces helps the user to interact with the AMAS. More specifically under certain conditions an Interface Agent can "program itself". That is it can acquire the knowledge it needs to assist its user.

The UI Agent is in fact oriented to learn from users and the system itself. Obviously the UI Agent has its own knowledge base.

To build another intelligent component of the AMAS low level, one needs to define the Expert System (ES) Agent which corresponds to an Expert System. For instance, the number of breeding sites with malaria vectors is generally related to the amount of rainfall for most of the vector species; but excessive rains cause flushing, thus killing immature stages. Considering the above close association of vector biology (DBMS Agent) with ecological parameters, rainfall, soil, altitude and temperature (GIS Agent), the ES Agent can then be required.

Then ES Agent is a rule-based system and contains in its knowledge base some of the following rules:

1) If zone is peripheral and habitat is not planned then mosquito breeding is favorable.

2) If zone is urban and habitat is planned then mosquito breeding is unfavorable.

3) If water is stagnant and temperature is high then mosquito breeding is favorable.

4) If mosquito breeding is favorable and plasmodium parasites in human population then malaria transmission is active.

5) If rainfall is weak and mosquito breeding is favorable then there is a season of abundance.

6) If soil is clayey and altitude is low then mosquito breeding is favorable.

7) If soil is sandy and rainfall is weak then water is not stagnant.

8) If water is not stagnant and temperature is high then mosquito breeding is unfavorable.

9) If rainfall is heavy and mosquito breeding is unfavorable then there is not a season of abundance.

10) If temperature is low and duration of parasite development in mosquitoes is long then malaria transmission is limited.

11) If habitat is traditional and mosquito breeding is favorable then mosquito biting rate is high.

12) If habitat is modern and mosquito breeding is unfavorable then mosquito biting rate is low.

13) Etc....

The ES Agent intends particularly to determine the possible existence of lairs in the various localities not covered in the study. Considering that mosquitoes breed in ponds, or marshy areas, the presence of natural settings or households can be deduced in the ES Agent.

Two main agents are considered in the AMAS high level to constitute analysis agents.

- The Data Mining (DM) Agent can be seen as a sub-system of Weka [12], which is a collection of machine learning algorithms for data mining tasks. The algorithms can be applied to a dataset or called from a Java code. Weka contains tools for data processing, classification, regression, clustering, association rules, visualization. In the AMAS we are interested actually by the numeric prediction capacity offered by Weka.

- The Decision Support System (DSS) Agent contains a set of rules as the ES Agent. It can in fact deliver decisions according to the:

- information produced by the environment (political issues, experiences conducted):

- memory of the AMAS kept in the ontology;

- data processed in the AMAS.

### Application to our system

According to the former two definitions, our AMAS is made of typically adaptive agents because their functioning can be local for each level and can be integrated to the whole system in general. For instance:

a) The DBMS Agent executes queries dealing with some statistic indications of fundamental trends (COUNT, AVERAGE, SUM, MIN, MAX, etc).

b) The GIS Agent delivers graphical indications of fundamental trends.

c) The Expert System Agent can deduce knowledge from both DBMS Agent and/or GIS Agent that can serve to Data Mining Agent and Decision Support Agent.

d) The User Interface Agent launches and dispatches queries to the other agents or decide to reproduce itself by generating new subsets

### 2.3 -Agents communication

#### Agents communication in the AMAS low level

Nowadays, there is a considerable interest in establishing the cooperation of a GIS with a DBMS in order to meet the requirements of advanced applications. In general, there are two ways to go for establishing the cooperation of a GIS and a DBMS:

a) systems interoperability, i.e., coupling of pre-existing systems using linkage components;

b) systems integration, i.e., design of a new system from scratch, which fuses all desired capabilities of individual technologies.

We adopt in the AMAS the first alternative. Because, a major factor is the high cost of building new comprehensive software systems from scratch, particularly when users' expectations for functions are set by the commercially-available GIS, spreadsheets, statistical analysis, visualization, modeling systems, etc. In addition, these are sophisticated and complex systems, which are being continuously upgraded. Another consideration is the desirability of incorporating systems already in use in an organization and which represents a high investment in training, establishing workflows, database assembly, etc.

On the top of the couple (GIS, DBMS), we are going to build the ES. And so we create another dimension of interoperability. An ES here can be a generator like JESS (Java Expert System Shell) [13]. The great advantage is that in this level, Java code is also going to be present.

#### Agents communication in the AMAS high level

**Figure 1:**
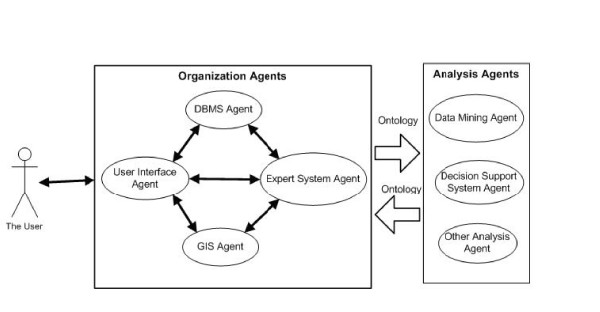
The AMAS architecture.

We recall that the DM Agent comprises algorithms and the DSS Agent is constituted of if-Then rules. Their communication is done usually in one direction. The DM Agent can send its results to the DSS Agent in order to achieve decisions. This is shown in Figure 1 below.

## 3-Results

### 3.1 AMAS low level

Let's assume that the user through the UI Agent wants to know some entomological parameters in some districts of Cameroon, a country in central Africa. We notice that in the system, data have just been collected in four houses per district. The system can deliver through the DBMS Agent and the GIS Agent the following results for Yaounde, and Edea, two towns in the south of Cameroon. We recall that Yaounde is the capital of Cameroon. Two districts of Yaounde are considered in the initial study: Nkol-Bisson and Essos. The district of Pongo is situated in Edea side. Results concern the A. gambiae vector and they are stemming from [14]. Table 1 presents these results.

**Table 1:** Entomological parameters of malaria transmission.

Parameter	House number				Avg
		
	1	2	3	4	
	Nkol Bisson				
Distance, m ^*a*^	50	70	300	350	190
Biting rate^*b*^	657	584	365	329	484
	Essos				
Distance, m^*a*^	10	70	150	250	120
Biting rate^*b*^	208	37	11	0	64
	Pongo				
Distance, m^*a*^	10	100	100	200	80
Biting rate^*b*^	1,049	223	198	0	368

^*a *^is the distance between house and breeding places

^*b *^is the number of bites per man per year

We have the following statements:

- Distance belongs to GIS Agent

- Biting rate belongs to DBMS Agent

- House number belongs to GIS Agent

The ES Agent is not necessary in this query because there is not derivation of knowledge. All the data are contained in the system.

One particular element in the AMAS low level is the ES Agent. The ES Agent serves to deduce knowledge namely when data are not contained in UI, DBMS, GIS Agents. This happens for localities where studies have not been carried out.

From our results and other studies the major vectors of malaria, differ greatly in their biology, breeding habitats and distribution. Looking to the vast areas, the reports on vector distribution, which is an essential component of selective malaria control strategy, are scanty. Different vector species establish their population at different heights where ecology is suited for their survival. Impermeable soil allows water stagnation and creates grounds for mosquito breeding and thus favors malaria.

Porous soil is devoid of stagnant water bodies, hence unfavorable for anopheles breeding. Longevity of vectors and the process of parasite development are sensitive to temperature. Vector species adapts to different temperature threshold depending on the area it occurs. Low temperature, when duration of parasite development in mosquitoes exceeds 30 days i.e. beyond average life span of mosquitoes limits active malaria transmission.

Here are some applications related to the AMAS low level:

• Point pattern can attempt to display the distribution of disease cases as data location (GIS Agent).

• Dynamic graph capability can highlight cases on maps allowing the eventual characterization of a region (GIS Agent).

• Overlying capacity can be used for identifying high risk areas and contaminated environmental factors (GIS Agent, ES Agent).

• Maps showing population density can be used to select sites for horizontal/vertical control strategy (DBMS Agent, GIS Agent).

• At the micro level, in a village malaria cases can be pinpointed to a specific coordinate. Control measures can be easily determined by overlying topological map (DBMS Agent, GIS Agent).

• Etc...

The AMAS in the low level will be answerable particularly some types of queries:

1) Queries answerable partially by at least two Agents

2) Ambiguous queries answerable by the DBMS Agent or the GIS Agent or the ES Agent or the UI Agent

3) Queries difficult to be answerable

Ambiguous queries should be answered by reproduction of the User Interface Agent. The User is invited by the User Interface Agent to dialogue in the natural language to precise the query.

When a query is difficult to be answerable in the low level, the solution might be delivered in the high level.

### 3.2-AMAS high level

Analysis Agents are involved in the satisfaction of fundamental trends. They are: the DM Agent and the DSS Agent etc.

Our Analysis Agents can respond to the following fundamental trends:

1) They can help as a decision support system (DSS Agent) in the mosquito and malaria control.

Decision making process under uncertainty is largely based on application of statistical data analysis for probabilistic risk assessment of decision. The DSS Agent analyses data found in the system and uses the interpreted results.

For example, in the locality of Simbock (300 habitants) situated 2 km from Yaounde, a study was conducted [15]. The DSS agent is capable to deliver the following results on malaria transmission, as presented in Table 2.

**Table 2:** Malaria transmission in Simbock, a locality of South Cameroon

Malaria Vectors	Malaria Transmission
*A. gambiae*	23.8%
*A. funestus*	26.8%
*A. moucheti*	39.2%
*A. nili*	10.2%

2) They can equally help in predicting (DM Agent).

In this way, malaria epidemics can be predicted in a particular locality given that the system is constantly enriched for every locality with information on rainfalls, temperatures, hygiene practices, etc.

The user is generally able to have results on some aspects of the fight against the disease through the AMAS high level. According to human beings tasks, the DSS Agent is able to compute the following measurements about the resistance of *A. gambiae *in regard with 3 types of insecticides in some localities of south Cameroon. Results are given in Table 3.

**Table 3:** Knockdown times and mortality rates for *A. gambiae*.

Sites	Insecticides	N	KdT_50 _[95% CI]	Mortality (%)
Foumbot	4% DDT	162	No Kd	6.8
	1% Permethrin	159	61.0 [55.3–75.2]	54.7
	0.05% Deltamethrin	165	22.5 [21.7–23.3]	87.9
Douala	4% DDT	100	58.0 [53.3–64.7]	32.3
	0.05% Lambdacyhalothrin	99	11.7 [11.0–12.3]	90.9
	0.05% Deltamethrin	100	7.3 [6.7–7.8]	100
Campo	4% DDT	157	29.1 [21.8–33.6]	96.8
	1% Permethrin	159	10.0 [8.4–11.5]	99.4
	0.05% Deltamethrin	158	8.4 [5.8–10.7]	100

N = Sample size; KdT_50 _= Knockdown time (in minutes) for 50% of mosquitoes; 95% CI = 95% Confidence Interval; Mortality = mortality rate 24 hours post exposure

From table 3, it is possible to have a huge difference in susceptibility (mortality) of mosquito population to the same Insecticide (DDT in Foumbot and in Campo), for the frequency of resistance gene in Foumbot compared to Campo [16].

A few obvious applications in the high level are the following:

• Pattern analysis could be used to describe epidemics diffusion (DM Agent).

• The vector data aids in analysis of disease diffusion pattern and health care facility flow (DM Agent).

• Population density map can also help in restructuring parasite control component by defining the catchments area of clinical facility. Also this information is useful for planning projected resource needs and the distribution/requirement of satellite clinical facilities (DSS Agent).

• Etc.

### 3.3-Ontology

**Figure 2:**
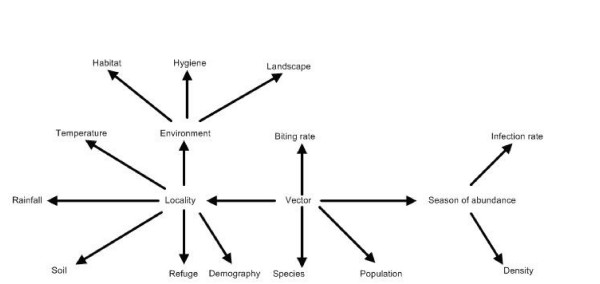
A part of ontology.

The ontology [17] is responsible of mapping concerned data from organization agents to analysis agents. It is in general the memory of the AMAS. We know the vocabulary of involved terms, and the specification of their meaning in the AMAS. We now need to indicate how concepts are inter-related. We get the representation of a part of our ontology in Figure 2.

The ontology is more complex than the illustration of figure 2. It really contains all entities handled by agents. What is represented here is just an initial schema of the ontology which is yet to be implemented. It is characterized by its evolutivity since it is enriched often by new terms. This is the case where new rules containing new attributes are introduced in the AMAS. For that purpose the ontology is monitored to learn from users and from agents. Protege2000 is the tool we are going to use to design the ontology [18].

#### Applications

A same question to be performed may use the ontology by crossing the AMAS two levels, so that it is answerable both by the low and the high levels. For instance let's look at the query expressed by the user through the UI Agent in the following terms: In what localities the danger of malaria epidemic is it true? The UI Agent may transform that query through its learning mechanism as follows: In what localities factors contributing to occurrence of malaria do exist? At least three factors can be returned: vector density, man biting, entomological inoculation rate (EIR).

The factors vector density and man biting rate are delivered in the AMAS low level. Now let's examine the EIR factor. This term is absent from the AMAS low level according to the ontology. The extension of the AMAS is necessary to find that factor. It depends on two attributes: man biting rate and infection rate. The DM Agent is the one that is aimed to evaluate this factor. Sometimes answers to queries may be more elaborated in the AMAS high level. So the fuzzy logic rules combined with the ontology may be used [19].

EIR is a common estimate of malaria transmission intensity. Here are EIR results in localities of Nkol-Bisson, Essos and Pongo through the DM Agent ability. This can help to predict risks of disease. Table 4 shows it.

**Table 4:** Entomological parameter(inoculation rate) of malaria transmission.^*c *^Entomological inoculation rate(number of infective bites per man per year)

Parameter	House number				Avg
		
	1	2	3	4	
	Nkol-Bisson				
Inoculation rate^*c*^	48.8	39.6	24.8	22	32.8
	Essos				
Inoculation rate^*c*^	43.2	6.8	2	0	13
	Pongo				
Inoculation rate^*c*^	86.3	18.3	16.3	0	30.2

**Figure 3:**
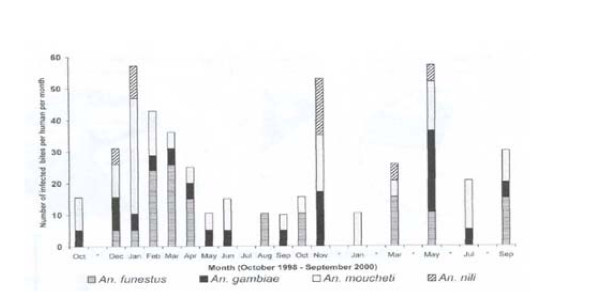
Monthly EIR for each vector species in Simbock.

In the locality of Simbock the DSS Agent may graphically show in Figure 3 that malaria transmission is perennial throughout the year, with seasonal variation, in terms of intensity and implication of the different vector species. Here, the ontology indicates that the factors intensity and implication of the different vectors are not mentioned in the AMAS low level. Here is Figure 3.

## 4-Discussion

The AMAS designed in this work is a step for a surveillance and a control system implementation, where some 20 localities in Cameroon have been studied. The main advantage of our system when compared to the existing situation is that one can get an overview of malaria transmission in a large number of localities without conducting studies in each of them. This is very important because for instance, high variations can be observed in malaria transmission depending on the year, or between villages few kilometers apart. The other big advantage is that all information system components are well highlighted to perform tasks only under their responsibility.

Particularly, one can study malaria dynamics both in space and time and relate the increase in malaria incidence to specific environment. This is undertaken in the AMAS low level. In the high level, one can study the impact of some specific environment on the community within its impact zone. Then the capabilities of the AMAS can be applied to a variety of planning, administrative and decisional activities for a health policy purpose.

## 5-Conclusion

We have justified the claim that agent-based computing has the potential to provide a powerful suite of metaphors, concepts and techniques for conceptualizing, designing and implementing complex (distributed) systems. Our choice lies on an AMAS. However if this potential is to be fulfilled, we have now to set up in the future the following needs of the system:

• Sharing knowledge inside and outside agents. We are going to put accent on the way agents are supposed to share knowledge with themselves and with the others. The multiplication of knowledge bases in the system demands the coordination of all them.

• All these requirements will be for enabling heterogeneous agents to inter-operate in open environments where coupling agents play a very big role.

Technically, JADE (Java Agent DEvelopment) framework is a good support on which the AMAS can be implemented [20]. Many issues related to our work can be handled by JADE modules: DF (Directory Facilitor), ACC (Agent Communication Channel), AMS (Agent Management System).

## Competing interests

The author(s) declare that they have no competing interests.

## Authors' contributions

GK is the supervisor of this work; he designed the ontology and the AMAS. AY took care of building and interoperability issues of agents. BN linked computing techniques with the entomological domain by implementing the system. JE worked on all aspects dealing with the vector biology and control, the parasite resistance, the expert system agent availability. FS was involved in malaria transmission and epidemiology problems.

## Pre-publication history

The pre-publication history for this paper can be accessed here:


